# The effects of sous-vide, microwave cooking and stewing of goose meat on fatty acid profile and lipid indices

**DOI:** 10.1016/j.psj.2022.102337

**Published:** 2022-11-12

**Authors:** Monika Wereńska, J. Wołoszyn, A. Okruszek, W. Marcinkowska, G. Haraf

**Affiliations:** Department of Food Technology and Nutrition, Wroclaw University of Economics and Business, Wroclaw 53-345, Poland

**Keywords:** goose meat, sous-vide, stewing, microwave cooking, fatty acid profile

## Abstract

The effect of sous-vide (**S-V**), microwave cooking (**M**) and stewing (**S**) on the fatty acid profile and lipid indices of goose meat was investigated. The research material involved breast muscles (24 with skin and 24 without skin) cut from carcasses of 17-week-old females “Polish oat geese.” Gas chromatography was used to establish the fatty acid profile and lipid indices were calculated. The kind of heat treatment and the type of goose meat (muscles with- and without skin) affected the fatty acid profile and lipid indices. The sum of SFA was higher in S-V, M, and S samples for both kinds of meat than in raw ones. The cooked samples with skin had a lower percentage of Ʃ SFA than the skinless meat. S-V and M cooking (for meat with skin) caused an increase, while in the case of S heating (for both kinds of meat) there were no significant differences in Ʃ MUFA compared to raw samples. The S-V, M, and S meat with skin was characterized by a higher value of Ʃ MUFA than skinless ones. The Ʃ PUFA was lower in S-V and M than in raw meat, wherein this decline was higher for M ones (for both kinds of meat). The M meat with skin had the lowest and S without skin the highest share of Σ PUFA. Heat treatment caused an increase in Σ PUFA *n-6/n-3* ratio, the lowest value was shown by the S-V muscles. Sous-vide cooking was more beneficial for consumers than the remaining methods in terms of Σ DFA/Σ OFA, Σ UFA/Σ SFA, NVI, health-promoting index (**HPI**), inflammatory biomarker indexes, and Σ SFA, Σ OFA, Σ UFA, Σ DFA values for meat with skin (but not all of these were significant). In turn, stewing of meat without skin was more favorable than S-V and microwave cooking in relation to indexes such as: Σ DFA/Σ OFA, Σ UFA/Σ SFA, Σ PUFA/Σ SFA, PI, UI, AI, TI, HPI.

## INTRODUCTION

Heat treatments are usually applied to meat prior to consumption and they are normally differentiated by the means of heat transfer. Regardless of the method of heat transfer in each case, cooking causes different changes in the nutritional value of meat, functional, and sensory properties of meat and guarantees their microbiological safety. However, different cooking methods can cause undesirable changes, such as lipid oxidation, formation of heterocyclic aromatic amines, polycyclic aromatic hydrocarbons and other substances harmful for humans (Pathare and Roskilly, 2016; Modzelewska-Kapituła et al., 2019; Suleman et al., 2019). From this perspective, the heat processes which ensure meat safety and meet the demands of the consumers without compromising their nutritional value, are required. Recently, consumers are becoming increasingly aware of the relationship between the chemical composition of consumed food and human health. One of the basic ingredients of food that undergoes significant changes during the thermal processes are lipids. The composition of the lipids fraction is one of the main dietary aspects to be considered in relation to the risk of cardiovascular and other civilization diseases of the 21st century. The factors which implicated in the development of these diseases these related to the lipid fraction are among others: cholesterol-raising saturated fatty acids (**SFA**), thrombogenic SFA, *n-*6 polyunsaturated fatty acids (**PUFA**), some PUFA *n-*3 and monounsaturated fatty acids (**MUFA**). The Ʃ PUFA *n-6* / *n-3* ratio is also very important, as a high concentration of PUFA *n-6* has a prothrombogenic, while PUFA *n-3* has anticoagulant and anti-inflammatory effect (Ulbricht and Southgate, 1991; Conchillo et al., 2004). Likewise, the trans fatty acids (**TFA**), which are formed in food during different industrial processes, does not play a positive role in vital functions. On the contrary, the intake of TFA may harm human health. Additionally, the changes in the fatty acids (**FA**) composition of foods are reflected too in the values of such indicators as: nutritive value index (**NVI**), hypocholesterolemic/hypercholesterolemic index (Ʃ DFA/ Ʃ OFA), Ʃ UFA/ Ʃ SFA ratio, health-promoting index (**HPI**), atherogenic (**AI**) and thrombogenic (**TI**) indices, inflammatory biomarker (**IB**), cardiovascular biomarker (**CB**), peroxidisability (**PI**) and unsaturation (**UI**) indexes (Wood et al., 2004; Chen and Liu, 2020; Simopoulos, 2020). A good source of lipids for the human is among others chicken and turkey meat, which are the most popular in all world countries, and the meat of waterfowl (including the goose meat) which is consumed in much smaller amounts. It is well known that goose meat provides fat with a high content of MUFA with one of the highest Ʃ UFA/Ʃ SFA ratio among the various types of meat. In Poland, to source and sell this meat commercially are used White Kołuda geese, and they are called “Polish oat geese,” because the birds are fattened freely with oats in the last 3 weeks of rearing. Goose meat is characterized by a specific, delicate taste and aroma as well as desirable tenderness, and it can be prepared in a variety of ways to meet the consumers’ flavour preferences (Nowicka, 2018). The most representative heat treatment methods used in cuisine for goose meat are: water bath cooking, grilling, pan-frying (with and without fat or oil), deep-fat frying, stewing, oven and microwave cooking (Oz and Celik, 2015). The alternatives to these methods for goose meat may be sous-vide (**S-V**) cooking, as a result of which a product classified as "ready to eat" is created. Sous-vide cooking is a new variant cooking technique used normally to produce high-quality dishes in the food service sector. The technique called S-V is one of the most popular forms of LT-LT (low temperature-long time) cooking in bag, which applies the principle to vacuum packed meat with water, or steam as heating media. Long-time low-temperature S-V cooking has received increasing popularity as the modern way of cooking meat (Baldwin, 2012; Dominguez-Hernandez et al., 2018; Gómez et al., 2019; Gómez et al., 2020).

It should be noted that the S-V technique has been not yet tested in terms of the quality of lipids present in goose meat. Which is way, the our study focused in particular, on an analysis of the FA profile and lipid indices of goose meat subjected to S-V cooking and comparison with conventional methods such as stewing and microwave cooking. This work complements our previous research (Wołoszyn et al., 2020; Goluch et al., 2021; Wereńska et al., 2021) on the effect of various heat treatment methods on the quality of goose meat.

## MATERIAL AND METHODS

### Meat Samples

Material for the experiment were the breast muscles (**BM**) obtained from 17-week old “Polish oat geese.” The geese were kept in the same environmental conditions and fed in the same way (Wołoszyn et al., 2020). The birds were slaughtered in an industrial slaughterhouse according to EU regulations - Council Regulation EC No 1099/2009. The eviscerated carcasses were cooled at the temperature 4°C for 24 h and then, the muscles were cut out. The average weight for BM with skin and subcutaneous fat was 450 ± 17 g, and without skin 308 ± 24 g. The 24 BM with skin and subcutaneous fat and 24 without skin were investigated.

### Heat Treatments

Sous-vide, microwave cooking and stewing methods were tested. In our experiment any food additives were used. Six BM with skin and 6 without skin (total 12 BM) were used in each kind of cooking. The final end-point temperature for microwave cooking methods was 75°C. It was checked by placement in the centre of each muscle Teflon-coated thermocouple (Type T, Omega Engineering Inc., Stamford, CT) combined with a temperature recorder (VAS Engineering Inc., San Diego, CA). After the completed thermal process, the muscles were cooled to room temperature and placed in a refrigerator at 4°C for 24 h. After this time, the muscles were removed from the refrigerator, allowed to reach ambient temperature in an atmosphere of air (21°C, 3 h) and they were tested.

### Sous-Vide

For the S-V cooking method, the temperature of 70°C and time 4 h (240 min) was used. Before cooking, the BM were weighed and placed into a polyamide/polyethylene pouches (heat resistance of -40°C to+120°C; O_2_, N_2,_ and CO_2_ permeability of up to 50cm^3^/m^2^; water steam permeability up to 4g/m^2^ per 24 h). Total thickness of the bag was 92 μm, they were suitable for vacuum packaging machines. After packing, the pouches were heat-sealed using vacuum sealing machine (Profi Line 40+, Hendi, Robakowo, Poland) with extent of vacuum 99.6%. Thereafter, the bags with meat were submerged in the water bath (model SW 22, Julabo GmbH, Seelbach, Germany) preheated to 70°C. The heating time of 4h was applied once the core temperature of the muscles reached the temperature of 70°C. For BM with skin and without skin they were made 2 control probes (a hand-held thermometer was used - Thermometer, DT-34 with a probe, Termoprodukt, Bielawa, Poland). Immediately after the heat treatment, the samples were removed from the water bath, rapidly chilled with ice-cold water (2°C) for 1 h and put in chilled storage (at 4°C for 24 h).

### Stewing (S)

Before stewing, the BM were first pan-fried with goose lard (10g) for 1 min per side. The BM to be stewed were placed individually on a stainless-steel pan and added 250 mL of hot water (the muscles were covered with water), covered with a lid and kept on the cooker for approximately 1h 30 min (90 min).

### Microwave Cooking (M)

Microwave cooking was performed using Whirpool MWP 253 SX (Whirlpool EMEA, Poland) microwave oven equipped with a revolving plate. Each breast muscle was cooked at 700 W, for 8 min (2 heating cycles of 4 min on each side of the BM), to reach a final core temperature about 75°C, required to achieve a constant degree of the centre of samples.

### Fatty Acid Profile

For analysis the FA profile, the raw and cooked BM were ground separately (mesh size 3 mm), next the samples were homogenized in a T 25 homogenizer (Ika Ultra-Turrax Corp., Staufen, Germany). Total lipid of the fresh and cooked goose meat was extracted as described by Folch et al. (1957), using chloroform and methanol in a ratio 2:1 (v/v). The analyses were conducted in duplicate. An antioxidant (butylated hydroxytoluene, BHT) was added at a concentration of 0.001% to the chloroform: methanol fat extracts. The fat extracts were dried in a rotary evaporator under vacuum and the extracts were dried in a vacuum oven at 50°C, using phosphorus pentoxide as moisture absorbent. The extracted fat muscle was stored in a glass vial, (with push-in top) under a blanket of nitrogen (Falowo et al., 2017). Thereafter the saponification and transesterification were made. An aliquot (50 mg) of muscle lipids was mixed with 3 mL of methanolic sodium hydroxide (5M) solution and incubated in a water bath (at 55°C) for 30 min. After heating, the mixture was cooled down and 400 μL (of 5.0 μg/mL) of nonadecanoic acid (C19:0, Sigma, St. Louis, MO) as an internal standard and 3 mL boron trifluoride (14% BF_3_) reagent were added. The mixture was incubated in water bath (at 55°C) for 30 min. After that, 2 mL of hexane and 2 mL of saturated sodium chloride solution were added and the mixture was centrifuged. In order to separate the FA, the supernatant was analysed by the gas chromatography technique (Campo et al., 2013; Li et al., 2016). Gas chromatography analysis of fatty acid methyl esters (**FAME**) were made in a Chromatograph (model 7890A, Agilent Tech., Santa Clara, CA) equipped: an automatic sampler (Agilent 7683), split injector (p/n 5183-4647), a fused silica capillary column (HP-88 J&W112-88A7 - 100 m length, 0.25 mm inner diameter - 0.20 μm film thickness) and a flame ionization detector. The carrier gas was helium. Parameters of analysis: injection of 1 μL, column rate constant flow 1.9 mL/min, split ratio 1:50, injection port temperature 240°C, flame ionization detector temperature 240°C, detector gases: helium make-up rate flow 30 mL/min; hydrogen 40 mL/min; air 450 mL/min. Oven temperature program was set at an initial temperature 120°C for 1min, then raised to 175°C (at 10°C/min) and held for 10 min, raised to 210°C (at 5°C/min) and held for 5 min and again raised to 230°C (at 5°C/min) and held for 5 min. Peaks were identified by comparison with the retention times of known standards. There were used Supelco 37 FAME Mix C 4-C 24 component (Sigma-Aldrich, St. Louis, MI). The FA were quantified in relation to the internal standard, nonadecanoic acid. The FA were expressed as a percentage of the sum of detected FAME (g/100g FAME), assuming direct proportionality between peak area and FAME weight. The analyses were conducted in duplicate.

### Calculation of Lipid Indices

The fatty acid profile was used to determine several nutritional parameters of lipids in goose BM. They were calculated the following lipid indices:

- Ʃ PUFA *n-6*/*n-3;*
- Ʃ PUFA/Ʃ SFA;
- Ʃ UFA/Ʃ SFA;
- NVI (Nutritive Value Index) = (C 18:0 + C18:1)/C 16:0 (Chen et al., 2016);
- AI (Atherogenic Index) = (C 12:0 + 4 × C 14:0 + C 16:0)/Σ UFA (Ulbricht and Southgate, 1991);
- TI (Thrombogenic Index) = (C 14:0 + C16:0 + C18:0)/[(0.5 × Σ MUFA) + (0.5 × Σ PUFA *n-6*) + (3 × Σ PUFA *n-3*) + (Σ PUFA *n-3*/Σ *n-6*)] (Ulbricht and Southgate, 1991);
- Ʃ OFA= (C14:0 + C16:0) dietary FA having an undesirable hypercholesterolemic effect in humans (Janiszewski et al., 2016);
- Ʃ DFA= (Σ MUFA + Σ PUFA + C18:0) dietary FA having a desirable neutral hypocholesterolemic effect in humans (Janiszewski et al., 2016);
- Ʃ DFA/Ʃ OFA;
- IB (Inflammatory Biomarker) = C20:4n-6/C20:5n-3 (Tutino et al., 2019);
- CB (Cardiovascular Biomarker) (sum of eicosapentaenoic and docosahexaenoic acids: EPA + DHA) (Sonnweber et al., 2018; Chen and Liu, 2020);
- HPI (Health-Promoting Index) = Σ UFA/[C 12:0 + (4 × C 14:0) + C 16:0]) (Chen and Liu, 2020);
- UI (Unsaturation Index) = 1 × (% monoenoics) + 2 × (% dienoics) + 3 × (% trienoics) + 4 × (% tetraenoics) +5 × (% pentaenoics) + 6 × (% hexaenoics) (Chen and Liu, 2020);
- Ʃ TFA (Trans Fatty Acids);
- PI (Peroxidisability Index) = (monoenoic acid × 0.025) + (dienoic acid × 1) + (trienoic acid × 2) + (tetraenoic acid × 4) + (pentaenoic acid × 6) + (hexaenoic acid × 8) ( Erickson, 1992).

### Statistical Analysis

The results were log-transformed to attain or approach a normal distribution, and next, a 2-way analysis of variance was used in the orthogonal system. The statistical significance of the differences between the averages of the groups was verified using Tukey's test, on the level of significance *P* ≤ 0.05, with the use of Statistica13.3 software (StatSoft Inc., 2019). The average values and their standard deviations were presented in the tables.

## RESULTS

### Fatty Acid Profile

The fatty acid profile (% of total FA) in raw and cooked (M, S-V, S) geese BM (with and without skin) was presented in Table 1, Table 2, Table 3, while lipid indices were listed in Table 4. In both type of raw and cooked muscles the percentage of individual groups of FA was as follows: MUFA>SFA>PUFA. The major FA in the raw and cooked muscles were C18:1cis (41.74-45.27; 42.88-47.83% respectively), C16:0 (20.30−22.38; 22.08−24.10% respectively), C18:2 *n-6* (14.13−15.24; 12.47−15.74% respectively) and C18:0 (4.70−4.94; 6.17−7.52% respectively). Both, kind of goose meat (*P* = 0.001−0.023 depending on the fatty acid) and the type of heat treatment (*P* = 0.001−0.028 depending on the fatty acid) affected the fatty acid profile. There was no effect of muscle type on the percentage of: C17:0 (*P* = 0.609), C16:1 (*P* = 0.078), C20:1 (*P* = 0.659) FA. There was no interaction between the type of meat × heat treatment for C17:0 (*P* = 0.943), C18:1 *trans* (*P* = 0.083), C18:1 *cis* (*P* = 0.246), C20:5 *n-3* (*P* = 0.192).

**Table 1:** Monounsaturated fatty acid (MUFA) profile of raw and cooked goose meat (% of total fatty acids) (n = 6 breast muscles with skin and n = 6 without skin for each kind of heat treatment).

Parameters	Meat	Raw meat (R)	Heat treatment			Total	*P-value***(***P* ≤ 0.05)		
			Microwafe (M)	Sous vide (SV)	Stewing (S)		Meat (M)	Heat treatment (T)	M x T
C 16:1	Without skin	3.40^a^ ± 0.12	y3.06^b^ ± 0.10	3.26^ab^ ± 0.27	2.79^c^ ± 0.15	3.13 ± 0.28	0.078	0.001	0.009
	With skin	3.30^a^ ± 0.18	x3.40^a^ ± 0.21	3.24^a^ ± 0.16	2.88^b^ ± 0.19	3.21 ± 0.27			
	Total	3.35^a^ ± 0.16	3.23^a^ ± 0.24	3.25^a^ ± 0.21	2.85^b^ ± 0.17				
C 18:1 *trans*	Without skin	1.81^b^ ± 0.01	2.31^a^ ± 0.04	1.81^b^ ± 0.03	1.74^c^ ± 0.03	1.92 ± 0.23	0.020	0.001	0.083
	With skin	1.77^bc^ ± 0.03	2.31^a^ ± 0.02	1.79^b^ ± 0.02	1.74^c^ ± 0.03	1.90 ± 0.24			
	Total	1.79^b^ ±0.03	2.31^a^ ± 0.03	1.80^b^ ± 0.03	1.74^c^ ± 0.03				
C 18:1 *cis*	Without skin	y41.74 ± 1.60	y43.89 ± 3.13	y42.88 ± 2.24	y43.23 ± 1.08	y42.94 ± 1.88	0.001	0.009	0.246
	With skin	x45.27^b^ ±0.75	x47.14^a^ ± 2.42	x47.83^a^ ± 1.48	x45.62^ab^ ± 2.00	x46.46 ± 2.00			
	Total	43.51^b^ ± 2.19	45.52^a^ ± 2.74	45.36^a^ ± 3.15	44.42^ab^ ± 1.98				
C 20:1	Without skin	x0.10a ± 0.02	0.09^ab^ ± 0.01	0.08^b^ ± 0.01	0.08^b^ ± 0.01	0.09 ± 0.02	0.659	0.028	0.009
	With skin	y0.08 ± 0.01	0.09 ± 0.02	0.09 ± 0.02	0.08 ± 0.01	0.09 ±0.01			
	Total	0.09 ± 0.02	0.09 ± 0.01	0.09 ± 0.02	0.08 ± 0.01				
C 24:1	Without skin	0.17^c^ ± 0.02	x0.20^ab^ ± 0.02	x0.21^a^ ± 0.02	x0.18^bc^ ± 0.01	x0.19 ± 0.02	0.001	0.004	0.001
	With skin	0.18^a^ ± 0.02	y0.17^ab^ ± 0.01	y0.16^ab^ ± 0.02	y0.15^b^ ± 0.02	y0.16 ± 0.02			
	Total	0.18^ab^ ± 0.02	0.18^a^ ± 0.04	0.19^a^ ± 0.05	0.16^b^ ± 0.04				
Ʃ MUFA	Without skin	y47.23 ± 1.61	y49.56 ± 2.01	y48.23 ± 2.01	y48.02 ± 1.05	y48.26 ± 1.85	0.001	0.007	0.286
	With skin	x50.60^b^ ± 0.88	x53.11^a^ ± 2.38	x53.11^a^ ± 1.37	x50.47^b^ ± 1.82	x51.82 ± 2.08			
	Total	48.91^c^ ± 2.15	51.34^a^ ± 2.81	50.67^ab^ ± 3.02	49.24^bc^ ± 1.93				

a-c Different letters in rows means statistically significant differences between group average, including thermal treatment (*P* ≤ 0.05).

x-y Different letter in columns means statistically significant differences between group average, including kind of meat (*P* ≤ 0.05).

In preventing cardiovascular disease (**CVD**), it is advantageous to consume a food including MUFA (especially C18:1 *cis*), which increases the activity of low-density lipoprotein receptors and decreases triglycerides and the cholesterol concentration in serum (Kien et al., 2014; Li et al., 2016). The most abundant group of FA present in raw and cooked BM was Ʃ MUFA, the proportion of which was in the range of 47.23 to 50.60% and 48.02 to 53.11% (respectively), depending on the type of meat and heat treatment methods (Table 1). Generally it was no interaction between the type of meat × heat treatment (*P* = 0.286). There were no significant differences (*P* ≤ 0.05) in percentage of Ʃ MUFA in M, S-V and S meat without skin compared to raw sample. The M and S-V meat with skin had the same proportion of Ʃ MUFA (53.11%). Microwave and S-V (for meat with skin) caused a slight increase of Ʃ MUFA compared to raw meat (50.60%). In turn S meat showed proportion of Ʃ MUFA similar to raw material. The meat without skin characterized by a lower value (48.26%) of Ʃ MUFA than skin sample (51.82%) (*P* = 0.001). The dominant MUFA in all muscles were C 18:1 *cis*, and C 16:1 with variation among the investigated types of meat (*P* ≤ 0.05). All type of heat treatment caused a slight increase (by 2.1−4.6%) in the proportion of C 18:1 *cis* in relation to raw material (*P* = 0.001). The differences in percentage of C 18:1 *cis* for meat without skin were no significant, however for M and S-V samples with skin were significant (*P* ≤ 0.05) compare to raw samples. The meat without skin had a lower value (42.94%) of C 18:1 *cis* than skin sample (46.46%) (*P* = 0.001). Comparing the heat treatment methods, it was observed decrease (by 2.9 for S-V, 3.5 for M and 14.9% for S) in percentage of C 16:1, and only in case S samples it was significant difference compared to raw ones (*P* = 0.001). The skin and without skin meat had similar percentage of C 16:1 (*P* = 0.078). In both kinds of meat, microwave cooking resulted a significant increase in C 18:1 *trans,* whereas stewing decrease compared to raw samples. The higher proportion of C 18:1 *trans* was produced during microwave than during S-V cooking and stewing. A significant increase in C 24:1 was observed for skinned meat subjected to microwave and S-V cooking (Table 1). It was no differences in proportion of C 24:1 for both kinds of meat. The sum of SFA was significantly higher in the, M, S-V and S samples (31.12, 30.33, 29.68% respectively) than in raw (26.72%) meat. Those changes in cooked meat might be explained by the fact that SFA are largely represented in neutral lipids and are prone to migration (Juárez et al., 2010). Of all the heat treatment methods, meat with skin had a lower percentage (28.44%) in the Ʃ SFA compared to the skinless ones (30.48%). Probably this may be caused by the more cooking losses compared to meat with skin. The lowest value of Ʃ SFA was established for S samples without skin and the highest for both type of M meat (*P* = 0.001). In general, the highest increase in the percentage of Ʃ SFA was caused in microwave (by 16.5%), next by S-V cooking (by 13.5%) while the lowest, stewing (by 11.1%) (*P* = 0.001) (data calculated on the basis of Table 2). The higher Ʃ SFA percentage in the heat-treated both types of meat was explained by the increase mainly of C 18:0 (stearic acid). Stearic acid is thought to be neutral with respect to atherogenicity but on the other hand instead considered to be thrombogenic. It does not raise plasma low-density lipoprotein cholesterol (**LDL-C**), C 18:0 may have some beneficial effects in regulating mitochondrial morphology and function, though these mechanisms are still being studied (Senyilmaz-Tiebe et al., 2018). Microwave cooking caused (for meat with and without skin) the greatest increase in the C 18:0 percentage (by 29.5% and 60% respectively) in relation to the value for raw meat. For all methods the meat without skin showed the higher percentage of C 18:0 compared to BM with skin, the opposite of that for raw meat. There was an increase of the C 16:0 (palmitic acid) proportion in M (by 6.5%) and S-V (by 7.2%) for BM without skin and in M (by 14.7%), S-V (by 8.8%), S (by 10.30%) muscles with skin compared to the raw meat (data calculated on the basis of Table 2). There was no significant difference in proportion of C 16:0 in S meat without skin in relation to raw material (P≤0.05). The S total breast samples had a significantly lower percentage of C 16:0 than M meat and similar to S-V ones. The C 14:0 (myristic acid), which is responsible for human hypercholesterolemia, was detected for stewing, microwave and S-V cooking at a low proportion in the studied samples (0.40−0.52% for meat without skin and 0.36−0.45% for meat with skin). The S muscles without skin were characterized by a lower percentage of C14:0 than raw meat, whereas the M and S-V were similar to raw ones (*P* ≤ 0.05). For skin meat, there were no significant differences in the percentage of C14:0 between all heat treatment methods and raw sample (Table 2). From a nutritional point of view it was very advantageous, demonstrating a positive factor in their consumption. In our study, the share of Σ PUFA varied from 14.80% in M meat with skin to 18.76% in S samples without skin. Generally, Ʃ PUFA was significantly lower in M and S-V and higher in S samples than in raw meat. Wherein this decline was by 16.7 and 7.1% for M and S-V (respectively), and an increase by 2.2% for S samples (*P* = 0.001) (data calculated on the basis of Table 3). For both kind of meat, the highest decrease compared to raw meat in Ʃ PUFA was stated for M meat without skin (by 20.11%) and the lowest for S meat without skin (by 3.2%). Among Σ PUFA, the main PUFA *n-6* fatty acid in all samples was C 18:2 *n-6* followed by C 20:4 *n-6*. The lower Ʃ PUFA percentage observed in M and S-V meat was explained by a decrease stated mainly in the proportion of individual PUFA such as: C 18:3 *n-3*, C 20:4 *n-6*, C 18:2 *n-6.* The higher percentage of Ʃ PUFA in S samples caused probably increase mainly in C 18:2 *n-6*. It is known that oxidative modification of C 18:2 *n-6*, which also takes place in the heating process, increases the atherogenicity of LDL-C. The lowest decrease of Ʃ PUFA was presented by C 20:4 *n-6* in S-V muscles with skin (by 0.8%), and the highest by C 18:3 *n-3* in M samples without skin (by 39.1%) (data calculated on the basis of Table 3). This high percentage decrease in C 18:3 *n-3* was an undesirable phenomenon, because in the animal body it is converted to a series of longer chain PUFA of which the most important for human are C 20:5 *n-3* (EPA) and C 22:6 *n-3* (DHA) (Ulbricht and Southgate, 1991). In our study the major PUFA *n-3* were the C 18:3 *n-3* and C 20:5 *n-3*, which did show significant differences between both type of muscles and heat treatment methods (Table 3). Losses of C 18:2 *n-6* in M were higher (by 11.7 for meat with skin and by 16.01% for meat without skin) than those of S-V meat, too (by 2.0 for meat with skin and by 4.6% for meat without skin). For both kinds of meat and M, S-V methods, it was observed that the decrease (in relation to raw meat) in Ʃ PUFA *n-3* was higher (by 36.2; 25.5% respectively) than in Ʃ PUFA *n-6* (by 14.4; 4.9% respectively) (data calculated on the basis of Table 3). In the conducted study, the changes obtained in FAs were likely due to the higher susceptibility of PUFA to oxidative degradation.

**Table 2:** Saturated fatty acid (SFA) profile of raw and cooked goose meat (% of total fatty acids) (n = 6 breast muscles with skin and n = 6 without skin for each kind of heat treatment).

Parameters	Meat	Raw meat (R)	Heat treatment				*P-value***(***P* ≤ 0.05)		
			Microwafe (M)	Sous vide (SV)	Stewing (S)	Total	Meat (M)	Heat treatment (T)	M x T
C 14:0	Without skin	x0.54^a^ ± 0.05	0.50^a^ ± 0.11	x0.52^a^ ± 0.06	x0.40^b^ ± 0.03	x0.49 ± 0.08	0.001	0.001	0.031
	With skin	y0.36 ± 0.03	0.45 ± 0.11	y0.41 ± 0.07	y0.36 ± 0.05	y0.40 ± 0.08			
	Total	0.45^a^ ± 0.10	0.47^a^ ± 0.11	0.46^a^ ± 0.08	0.38^b^ ± 0.04				
C 16:0	Without skin	x22.38^c^ ± 0.42	23.84^ab^ ± 0.90	x24.10^a^ ± 0.78	22.89^bc^ ± 0.76	x23.30 ± 0.99	0.001	0.001	0.005
	With skin	y20.30^b^ ± 0.56	23.29^a^ ± 0.95	y22.08^a^ ± 1.07	22.39^a^ ± 1.07	y22.01 ± 1.42			
	Total	21.34^c^ ±1.18	23.57^a^ ± 0.94	23.09^ab^ ± 1.38	22.64^b^ ± 0.93				
C 17:0	Without skin	x0.12 ± 0.02	0.13 ± 0.02	x0.13 ± 0.03	x0.13 ± 0.03	x0.13 ± 0.02	0.609	0.001	0.943
	With skin	y0.09 ± 0.02	0.11 ± 0.04	y0.10 ± 0.03	y0.10 ± 0.03	y0.10 ± 0.02			
	Total	0.11 ± 0.02	0.12 ± 0.03	0.12 ± 0.03	0.11 ± 0.03				
C 18:0	Without skin	y4.70^b^± 0.33	x7.52^a^ ± 0.92	x7.10^a^ ± 0.36	x6.94^a^ ± 0.64	x6.56 ± 1.26	0.023	0.001	0.001
	With skin	x4.94^b^ ± 0.48	y6.40^a^ ± 0.78	y6.22^a^ ± 0.76	y6.17^a^ ± 0.77	y5.93 ± 0.90			
	Total	4.82^b^ ± 0.42	6.96^a^ ± 1.01	6.66^a^ ± 0.74	6.56^a^ ± 0.79				
Ʃ SFA	Without skin	x27.74^c^ ± 0.33	31.99^a^ ± 1.78	x31.85^ab^ ± 1.02	x30.35^b^ ± 1.13	x30.48 ± 2.07	0.001	0.001	0.001
	With skin	y25.69^b^^4^ ± 0.57	30.24^a^ ± 1.33	y28.81^a^ ± 1.28	y29.01^a^ ± 1.14	y28.44 ± 2.01			
	Total	26.72^c^ ± 1.15	31.12^a^ ± 1.77	30.33^ab^ ± 1.92	29.68^b^ ± 1.30				

a-c Different letters in rows means statistically significant differences between group average, including thermal treatment (*P* ≤ 0.05).

x-y Different letter in columns means statistically significant differences between group average, including kind of meat (*P* ≤ 0.05).

**Table 3:** Polyunsaturated fatty acid (PUFA) profile of raw and cooked goose meat (% of total fatty acids) (n = 6 breast muscles with skin and n = 6 without skin for each kind of heat treatment).

Parameters	Meat	Raw meat (R)	Heat treatment			Total	*P-value***(***P* ≤ 0.05)		
			Mikrowafe (M)	Sous vide (SV)	Stewing (S)		Meat (M)	Heat treatment (T)	M x T
C 18:2 *n-6*	Without skin	x15.24^b^ ± 0.38	x12.80^d^ ± 0.23	x14.54^c^ ± 0.28	15.74^a^ ± 0.46	x14.57 ± 1.18	0.001	0.001	0.001
	With skin	y14.13^b^ ± 0.29	y12.47^d^ ± 0.13	y14.42^c^ ± 0.32	15.59^a^ ± 0.52	y13.91 ± 1.20			
	Total	14.69^b^ ± 0.66	12.63^d^ ± 0.25	14.00^c^ ± 0.64	15.67^a^ ± 0.48				
C 18:3 *n-3*	Without skin	1.28^a^ ± 0.04	0.78^c^ ± 0.06	0.88^b^ ± 0.06	0.89^b^ ± 0.08	x0.96 ± 0.20	0.001	0.001	0.001
	With skin	0.77^b^ ± 0.05	0.67^c^ ± 0.09	0.75^bc^ ± 0.03	0.85^a^ ± 0.05	y0.76 ± 0.09			
	Total	1.03^a^ ± 0.27	0.73^c^ ± 0.10	0.82^b^ ± 0.08	0.87^b^ ± 0.07				
C 20:4 *n-6*	Without skin	x1.89^a^ ± 0.07	x1.34^d^ ± 0.02	x1.68^b^ ± 0.05	x1.47^c^ ± 0.09	x1.60± 0.22	0.001	0.001	0.001
	With skin	y1.22^b^ ± 0.06	y1.18^b^ ± 0.10	y1.23^b^ ± 0.07	y1.45^a^ ± 0.07	y1.27 ± 0.13			
	Total	1.56^a^ ± 0.35	1.26^c^ ± 0.11	1.46^b^ ± 0.24	1.46^b^ ± 0.10				
C 20:5 *n-3*	Without skin	x0.86^a^ ± 0.03	x0.48^c^ ± 0.04	x0.58^b^ ± 0.04	x0.55^b^ ± 0.04	x0.62 ± 0.15	0.001	0.001	0.192
	With skin	y0.78^a^ ± 0.04	y0.38^c^ ± 0.04	y0.51^b^ ± 0.03	y0.48^b^ ± 0.03	y0.54 ± 0.16			
	Total	0.82^a^ ± 0.06	0.43^c^ ± 0.07	0.55^b^ ± 0.05	0.52^b^ ± 0.05				
C 22:6 *n-3*	Without skin	0.12^a^ ± 0.01	0.09^b^ ± 0.01	0.11^a^ ± 0.01	x0.12^a^ ± 0.01	x0.11 ± 0.01	0.012	0.024	0.024
	With skin	0.11 ± 0.01	0.10 ± 0.01	0.10 ± 0.01	y0.10 ± 0.02	y0.10 ± 0.01			
	Total	0.11^a^ ± 0.01	0.10^b^ ± 0.01	0.11^ab^ ± 0.01	0.11^ab^ ± 0.02				
Ʃ PUFA *n-3*	Without skin	x2.26^a^ ± 0.07	x1.36^c^ ± 0.07	x1.57^b^ ± 0.07	x1.56^b^ ± 0.10	x1.68 ± 0.35	0.001	0.001	0.001
	With skin	y1.66^a^ ± 0.08	y1.15^c^ ± 0.08	y1.36^b^ ± 0.05	y1.43^b^ ± 0.06	y1.40 ± 0.20			
	Total	1.96^a^ ±0.32	1.25^c^ ± 0.13	1.46^b^ ±0.12	1.49^b^ ± 0.10				
Ʃ PUFA *n-6*	Without skin	x17.13^a^ ± 0.36	x14.14^c^ ± 0.23	x16.22^b^ ± 0.30	x17.21^a^ ± 0.52	x16.17 ± 1.31	0.001	0.001	0.001
	With skin	y15.35^b^ ± 0.32	y13.65^d^ ± 0.15	y14.64^c^ ± 0.29	y17.04^a^ ± 0.55	y15.17 ± 1.30			
	Total	16.24^b^ ± 1.47	13.90^d^ ± 0.31	15.43^c^ ± 0.86	17.12a ± 0.53				
Ʃ PUFA	Without skin	x19.39^a^ ± 0.39	x15.49^d^ ± 0.25	x17.79^c^ ± 0.33	x18.76^b^ ± 0.60	x17.86 ± 1.55	0.001	0.001	0.001
	With skin	y17.01^b^ ± 0.35	y14.80^d^ ± 0.20	y16.01^c^ ± 0.25	y18.45^a^ ± 0.59	y16.57 ± 1.41			
	Total	18.20^a^ ± 1.28	15.15^c^ ± 0.42	16.90^b^ ± 0.96	18.61^a^ ± 0.60				

a-d Different letters in rows means statistically significant differences between group average, including thermal treatment (*P* ≤ 0.05).

x-y Different letter in columns means statistically significant differences between group average, including kind of meat (*P* ≤ 0.05).

### Lipid Indices

In this study, the type of goose meat (*P* = 0.001, except Σ PUFA/Σ SFA, UI) and the kind of heat treatment (*P* = 0.001−0.002, except Σ UFA, NVI,) influenced the values of lipid indices. There were interactions between the type of meat × heat treatment for Σ OFA, Σ PUFA/Σ SFA, Σ PUFA *n-6/n-3*, NVI, PI, IB, UI,) indices (*P* = 0.001−0.04). The kind of heat treatment no influenced on Σ UFA percentage in goose meat compare to raw material. The BM without skin had lower proportion of Σ UFA than with skin ones (Table 4). Generally, the heat treatment caused a decrease in the Σ UFA/Σ SFA ratio for all samples. The Σ UFA/Σ SFA ratios accounted for 2.04 in M without skin to 2.41 in S-V meat with skin and were beneficial for human health (*P* ≤ 0.05). The meat without skin showed lower value than with skin ones (*P* = 0.001). Σ PUFA/Σ SFA is an index normally used to assess the impact of diet on cardiovascular health. It hypothesizes that all PUFA in the diet can depress LDL-C and lower levels of serum cholesterol, whereas all SFA contribute to high levels of serum cholesterol. Thus, the higher value of this ratio, the more positive the effect, this ratio above 0.45 is recommended (Chen and Liu, 2020). Foods with Σ PUFA/Σ SFA ratio below 0.45 have been considered undesirable for human diet. In our experiment, the Σ PUFA/Σ SFA ratios ranged from 0.49 to 0.63 depending on the kind of heat treatments. It was no significant differences between both kind of meat. The heat treatment meat was characterized by lower values of Σ PUFA/Σ SFA than raw ones. The most favorable Σ PUFA/Σ SFA was presented by the S skin samples (0.64). The Σ PUFA/Σ SFA ratios for both kinds of meat and for M, S-V, S techniques were higher than recommended and that means the better balance of FA in goose meat.

**Table 4:** Nutritional quality indices of the lipids in raw and cooked goose meat (n = 6 breast muscles with skin and n = 6 without skin for each kind of heat treatment).

Parameters	Meat	Raw meat (R)	Heat treatment				*P-value***(***P* ≤ 0.05)		
			Microwave (M)	Sous vide (SV)	Stewing (S)	Total	Meat (M)	Heat treatment (T)	M x T
Ʃ UFA (%)	Without skin	66.61 ± 1.68	y65.05 ± 1.88	y66.02 ± 2.15	y66.78 ± 1.14	y66.12 ± 1.80	0.001	0.117	0.297
	With skin	67.11 ± 0.87	x67.91 ± 2.26	x69.12 ± 1.43	x68.92 ± 1.51	x68.39 ± 1.65			
	Total	67.11 ± 1.39	66.48 ± 2.49	67.57 ± 2.38	67.85 ± 1.70				
Ʃ DFA (%)	Without skin	71.31 ± 1.71	72.57 ± 2.28	y73.12 ± 2.12	73.71 ± 1.42	y72.68 ± 2.03	0.001	0.002	0.881
	With skin	72.55^b^ ± 0.92	74.31^ab^ ± 2.17	x75.33^a^ ± 1.59	75.08^a^ ± 1.88	x74.32 ± 1.96			
	Total	71.93^b^ ± 1.47	73.44^ab^ ± 2.33	74.23^a^ ± 2.14	74.40^a^ ± 1.76				
Ʃ OFA (%)	Without skin	x22.92 ± 0.43	24.34 ± 0.97	x24.61 ± 0.81	23.29 ± 0.76	x23.79 ± 1.02	0.001	0.001	0.004
	With skin	y20.67 ± 0.56	23.74 ± 1.04	y22.50 ± 1.13	22.75 ± 1.10	y22.41 ± 1.47			
	Total	21.79^c^ ± 1.26	24.04^a^ ± 1.02	23.55^ab^ ± 1.45	23.02^b^ ± 0.95				
Ʃ DFA /Ʃ OFA	Without skin	y3.11 ± 0.13	2.99 ± 0.15	y2.98 ± 0.18	3.17 ± 0.13	y3.06 ± 0.16	0.001	0.001	0.068
	With skin	x3.51^a^ ± 0.14	3.14^b^ ± 0.22	x3.36^ab^ ± 0.23	3.31^ab^ ± 0.21	x3.33 ± 0.24			
	Total	3.31 ± 0.24	3.06 ± 0.20	3.17 ± 0.28	3.24 ± 0.19				
Ʃ UFA /Ʃ SFA	Without skin	y2.40^a^ ± 0.09	y2.04^c^ ± 0.13	y2.08^bc^ ± 0.13	y2.20^b^ ± 0.10	y2.18 ± 0.18	0.001	0.001	0.321
	With skin	x2.63^a^ ± 0.09	x2.25^b^ ± 0.16	x2.41^b^ ± 0.15	x2.38^b^ ± 0.12	x2.42 ± 0.19			
	Total	2.52^a^ ± 0.15	2.15^c^ ± 0.18	2.24^bc^ ± 0.22	2.29^b^ ± 0.14				
Ʃ PUFA /Ʃ SFA	Without skin	x0.69^a^ ± 0.02	0.49^d^ ± 0.03	0.56^c^ ± 0.02	0.62^b^ ± 0.04	0.59 ± 0.08	0.560	0.001	0.040
	With skin	y0.66^a^ ± 0.02	0.49^c^ ± 0.02	0.56^b^ ± 0.03	0.64^a^ ± 0.03	0.59 ± 0.07			
	Total	0.68^a^ ± 0.03	0.49^d^ ± 0.02	0.56^c^ ± 0.02	0.63^b^ ± 0.03				
Ʃ PUFA *n-6/n-3*	Without skin	y7.59^c^ ± 0.2	y10.45^b^ ± 0.52	10.38^b^ ± 0.43	y11.09^a^ ± 0.45	y9.88 ± 1.43	0.001	0.001	0.001
	With skin	x9.26^c^ ± 0.40	x11.96^a^ ± 0.79	10.77^b^ ± 0.58	x11.96^a^ ± 0.39	x10.99 ± 1.25			
	Total	8.43^c^ ± 0.91	11.20^a^ ± 1.02	10.57^b^ ± 0.53	11.52^a^ ± 0.61				
NVI	Without skin	y2.15 ± 0.10	2.26 ± 0.12	yy2.15 ± 0.15	2.27 ± 0.10	y2.21 ± 0.13	0.001	0.960	0.012
	With skin	x2.56 ± 0.11	2.40 ± 0.19	x2.54 ± 0.19	2.40 ± 0.20	x2.48 ± 0.18			
	Total	2.36 ± 0.23	2.33 ± 0.17	2.35 ± 0.26	2.33 ± 0.17				
AI	Without skin	x0.37^b^ ± 0.02	0.39^a^ ± 0.02	x0.40^a^ ± 0.03	0.37^b^ ± 0.02	x0.38 ± 0.02	0.001	0.001	0.135
	With skin	y0.32^b^ ± 0.01	0.37^a^ ± 0.03	y0.34^ab^ ± 0.03	0.35^ab^ ± 0.02	y0.35 ± 0.03			
	Total	0.34^c^ ± 0.03	0.38^a^ ± 0.03	0.37^ab^ ± 0.04	0.36^bc^ ± 0.02				
TI	Without skin	x0.59^c^ ± 0.02	x0.68^a^ ± 0.04	x0.67^a^ ± 0.03	x0.63^b^ ± 0.03	x0.64 ± 0.05	0.001	0.001	0.428
	With skin	y0.54^b^ ± 0.02	y0.62^a^ ± 0.05	y0.59^a^ ± 0.04	y0.58^ab^ ± 0.03	y0.58 ± 0.04			
	Total	0.57^c^ ± 0.04	0.65^a^ ± 0.06	0.63^ab^ ± 0.05	0.60^b^ ± 0.03				
PI (%)	Without skin	x32.64^a^ ± 0.51	x24.61^c^ ± 0.35	x28.57^b^ ± 0.60	28.83^b^ ± 0.98	x28.66 ± 2.95	0.001	0.001	0.001
	With skin	y27.36^a^ ± 0.68	y22.94^c^ ± 0.49	y25.03^b^ ± 0.27	28.01^a^ ± 0.79	y25.84 ± 2.11			
	Total	30.00^a^ ± 2.78	23.78^d^ ± 0.95	26.80^c^ ± 1.88	28.42^b^ ± 0.96				
HPI	Without skin	y2.72^ab^ ± 0.12	y2.52^b^ ± 0.16	y2.53^b^ ± 0.17	y2.73^a^ ± 0.12	y2.63 ± 0.17	0.001	0.001	0.118
	With skin	x3.11^a^ ± 0.11	x2.72^b^ ± 0.22	x2.92^ab^ ± 0.21	x2.90^ab^ ± 0.18	x2.91 ± 0.23			
	Total	2.91^a^ ± 0.23	2.62^c^ ± 0.21	2.73^bc^ ± 0.28	2.82^ab^ ± 0.17				
IB	Without skin	x2.20^b^ ± 0.11	2.80^a^ ± 0.21	x2.94^a^ ± 0.21	y2.71^a^ ± 0.22	x2.66 ± 0.34	0.001	0.001	0.001
	With skin	y1.56^c^ ± 0.09	3.15^a^ ± 0.44	y2.40^b^ ± 0.19	^x^3.03^a^ ± 0.28	y2.54 ± 0.69			
	Total	1.88^c^ ± 0.34	2.97^a^ ± 0.38	2.67^b^ ± 0.34	2.87^ab^ ± 0.30				
CB (%)	Without skin	x0.98^a^ ± 0.04	x0.58^c^ ± 0.03	x0.69^b^ ± 0.05	x0.66^b^ ± 0.04	x0.73 ± 0.16	0.001	0.001	0.632
	With skin	y0.89^a^ ± 0.05	y0.48^c^ ± 0.04	y0.61^b^ ± 0.03	y0.58^b^ ± 0.03	y0.64 ± 0.16			
	Total	0.93^a^ ± 0.06	0.53^c^ ± 0.06	0.65^b^ ± 0.05	0.62^b^ ± 0.05				
UI (%)	Without skin	x94.11a ± 1.84	85.83c ± 1.73	90.22b ± 2.44	91.50ab ± 1.68	90.42 ± 3.56	0.821	0.001	0.001
	With skin	y90.61^b^ ± 1.01	87.28^c^ ± 1.99	90.27^b^ ± 1.35	92.98^a^ ± 1.42	90.28 ± 2.49			
	Total	92.36^a^ ± 2.31	86.56^c^ ± 1.95	90.24^b^ ± 1.91	92.24^a^ ± 1.68				
TFA (%)	Without skin	1.81^b^ ± 0.01	2.31^a^ ± 0.04	1.81^b^ ± 0.03	1.74^c^ ± 0.03	1.92 ± 0.23	0.020	0.001	0.083
	With skin	1.77^bc^ ± 0.03	2.31^a^ ± 0.02	1.79^b^ ± 0.02	1.74^c^ ± 0.03	1.90 ± 0.24			
	Total	1.79^b^ ±0.03	2.31^a^ ± 0.03	1.80^b^ ± 0.03	1.74^c^ ± 0.03				

a-c Different letters in rows mean statistically significant differences between group average, including thermal treatment (*P* ≤ 0.05).

x-y Different letter in columns means statistically significant differences between group average, including kind of meat (*P* ≤ 0.05). Abbreviations: AI, atherogenic index; Σ DFA, Σ MUFA + Σ PUFA + C18:0; Σ OFA, C14:0 + C16:0; CB, cardiovascular biomarker; DFA/Σ OFA, hypocholesterolemic/hypercholesterolemic index; UFA, unsaturated fatty acids; HPI, health-promoting index; IB, inflammatory biomarker; NVI, nutritive value index; PI, peroxidisability index; TFA, trans fatty acids; TI, thrombogenic index; UI, unsaturation index.

The findings concerning the Σ PUFA *n-6*/*n-3* ratios in our investigation for all investigated samples were higher than the adequate value (<4). The values of the Σ PUFA *n-6/ n-3* ratios were in the range of 10.38-11.09 for muscles without skin and 10.77-11.96 for meat with skin, depending on the heat treatment method (P≤0.05) The Σ PUFA *n-6/n-3* ratios for raw material were lower than for cooked muscles. The lowest Σ PUFA *n-6/n-3* ratio was presented by S-V samples in comparison to M and S meat (*P* = 0.001). Skinned meat was characterized by a lower value of Σ PUFA *n-6/n-3* ratios than samples with skin (Table 4). Taking into account the value of Σ PUFA *n-6/n-3* in our experiment, the S-V cooking was the most appropriate methods of heat treatment for both type of meat. There were no significant differences in the Σ PUFA *n-6/ n-3* ratios between M and S meat (*P* = 0.001). Higher value of the NVI was shown by heat treatment samples with skin than those without skin. The highest value of NVI was characteristic for S-V meat with skin (2.54), but there were no significant differences between M and S samples. It was a consequence of similar values of C 18:0, C 18:1 *cis*, and C 16:0 among all cooked samples. Generally, the kind of heat treatment had no impact on value of NVI. PUFA differ in their antithrombogenicity activity, which is most pronounced in the PUFA *n-3*, especially EPA and DHA. They can reduce the risk of CVD, hypertension and inflammation. DHA is a critical component of the retina and the neuronal system and is involved in visual functioning and cognitive functioning in humans. Therefore, the Ʃ EPA and DHA is often named cardiovascular biomarker (**CB)** or omega-3 index (Sonnweber et al., 2018; Genova Diagnostics, 2021). In the present study the percentage of CB in goose muscles was very low, and varied from 0.48 % in M skin meat to 0.69% in S-V meat without skin. There were no significant differences between S-V and S samples (Table 4). Generally, the heat treatment lowered the proportion of CB compared to raw meat. The meat without skin showed higher values of CB than skin ones (*P* = 0.001). When analyzing the proportion of the hypocholesterolemic FA (DFA) of the investigated muscles, it was established no significant differences between raw and cooked samples without skin (Table 4). The S-V and S meat with skin had higher percentage of Σ DFA than raw sample. The ƩDFA represents 72.57 to 75.33% of total FA depending on the kinds of meat and heat treatment methods. We did not observe the effect of the heating method on the proportions of hypercholesterolemic acids (**OFA**) in both types of meat, however, we found a higher proportion of them in meat with skin. The highest percentage of Σ DFA and lowest of Σ OFA were shown for S-V meat with skin. The Σ DFA/Σ OFA ratio indicated the effects of specific FA on cholesterol metabolism and higher Σ DFA/Σ OFA values are considered more beneficial for human health. The Σ DFA/Σ OFA indexes ranged from 2.98 for S-V meat without skin to 3.36 for S-V meat with skin. None of the used thermal treatments significantly worsen the Σ DFA/Σ OFA ratio compared to raw meat. The atherogenicity, thrombogenicity indexes indicate potential for stimulating platelet aggregation (Ghaeni and Ghahfarokhi, 2013). Thus, the smaller the AI and TI values had greater protective potential for coronary artery disease. In terms of human health, the AI and TI indices, which are less than 1.0 and 0.5 respectively in the diet, are recommended (Fernandes et al., 2014). The cooked meat without and with skin showed an AI index in range of 0.37 to 0.40 and 0.34 to 0.37, respectively, and these results were lower than recommended (Table 4). The S-V sample with skin was characterized by the lowest values of the AI index, but there were no significant differences with M and S ones. The TI index was in the range of 0.63 to 0.68 for meat without skin and 0.58 to 0.62 for meat with skin, depending on used heat treatment method. The S sample with skin had the lowest TI index, but there were no significant differences between all 3 methods. All values of TI indices for both types of meat regardless of the heat treatment method were higher than the adequate, but they are very close to expected value. The meat without skin showed higher values AI and TI ratio than skin samples. The heat treatment caused an increase of TI indexes in comparison to raw meat and thus its deterioration (*P* = 0.001). The HPI is used to assess the nutritional value of dietary fat, which focuses on the effect of FAs composition on CVD. The products with the high value are assumed to be more beneficial to human health (Chen and Liu, 2020). The values of HPI in cooked meat was in range 2.52 to 2.73 for samples without skin and 2.72 to 2.92 for skin ones, depend on kind of heating process. The HPI index for meat without skin was lower than with skin. The lower value of HPI showed M and S-V than S meat without skin, there were no significant differences for M, S-V, S meat with skin. Generally, the goose meat subjected to 3 types of thermal treatment, achieved the HPI index satisfactory from the human healthy point of view. The value of IB index, termed the inflammatory biomarker depends on the content of C 20:4 *n-6* (AA) and C 20:5 *n-3* (EPA) acids in food. Because of C 20:4 *n-6* role in the inflammatory cascade and ability to induce oxidative stress, AA is a relevant factor in the pathogenesis of cardiovascular and metabolic diseases such as diabetes mellitus, non-alcoholic fatty liver disease, atherosclerosis, peripheral vascular disease, and hypertension EPA (*n-3*) and AA (*n-6*) both compete for use of the delta-5-desaturase enzyme to be synthesized. Increased dietary intake of animal fats alters fatty acid metabolism in favor of inflammation. There are many chronic diseases associated with elevations of this ratio including CVD, mood disorders, and cancer (Tutino et al., 2019). Therefore, this ratio the bigger the more dangerous. In our experiment IB indexes accounted for 2.40 in S-V to 3.15 in M meat with skin. The heat treatment caused increase IB indexes for all samples, regardless of the type of meat and the method of thermal processing. Thus, the increase of IB indexes significantly worsened the health values of the meat. The highest increase of IB in relation to raw meat showed M (by 101.9%), follow S (by 94.2%) and S-V (by 53.8%) meat with skin. The samples with skin had the lower value of IB than without skin, but IB increase was significantly greater and amounted to 62.8% compare to 20.9% for samples without skin (data calculated on the basis Table 4). In turn, the PI index represents the relationship between the fatty acid composition of a food products and its susceptibility to oxidation. The PI index is used to assess the stability of PUFA included in food products and to protect them from possible oxidation processes, but higher PI value is greater for the protective potential for coronary artery disease (Kang et al., 2005). In our study the cooked samples had a lower (23.78−28.42%) protective potential (PI) for coronary artery disease than raw (30.00%) ones (*P* = 0.001). This means that the all presented heat treatment methods reduced the PI value. The M meat with skin had the lowest PI index and S meat without skin the highest. This indicates a lowest susceptibility to auto-oxidation of FA in M meat with skin, but lower protective potential for coronary artery disease compared to the remaining ones, too. No significant differences in PI value were observed for S-V and S skinned meat (*P* ≤ 0.05). The meat without skin amounted higher PI value than with skin ones (*P* = 0.001) and thus, had a greater susceptibility to auto-oxidation of FA compared to heat-treated muscles with skin. The research showed that the S muscles were the most prone to auto-oxidation in comparison to other ones (*P* = 0.001). The UI indicates the degree of unsaturation in lipids and is calculated as the sum of the percentage of each unsaturated FAs multiplied by the number of double bonds within that FAs. This index indicates the impact of highly unsaturated FAs and does not ignore the impact of FAs that have a low degree of unsaturation. In general, the UI more comprehensively reflects the proportion of FAs with different degrees of unsaturation in the total FA composition of a food species (Johnson and Schaefer, 2006). Unsaturated FA are more heat-labile, and as the degree of unsaturation increases, usually they become less stable (Koubaa et al., 2012). The value of UI parameter in heat treatment meat ranged from 85.83% in M muscles without skin to 92.98% in S meat with skin. There were no significant differences between meat with skin and without skin for all cooking methods (*P* = 0.821). The M and S-V meat had lower (86.56%, 90.24% respectively) degree of unsaturation (UI) compared to S (92.24%), sample (*P* = 0.001). This could mean that these types (M and S-V cooking) of heat treatment caused less likely to oxidize the FA in goose meat.

## DISCUSSION

During heat treatment the fatty acid composition of lipids in meat may change. The mechanisms such as: water loss, lipid oxidation, diffusion exchange that occur during cooking, can lead to relative changes in some FAs proportion (Dal Bosco et al., 2001; Alfaia et al., 2010). The changes occurring in FAs during thermal process also depends on the type of lipid fraction (e.g. neutral, polar), too. The most heat-unstable group of FAs with the greatest changes due to the high degree of unsaturation are PUFA acids. Alfaia et al. (2010;) and Larsen et al. (2010) pointed that PUFA content is most prone to changes due to their primary location in phospholipids. On account of significant diversity research material, its size, weight, application of different heating techniques and parameters (e.g., time, temperature, heating rate, environment, temperature in the center of sample), discussing and comparing the results of different studies is often very difficult. The effects of S-V and steam cooking on fatty acid composition, in beef semimembranosus muscle were evaluated in the study of Modzelewska-Kapituła et al. (2019). The S-V cooking was conducted at 60°C for 4 h. As in our study, the predominant FAs in the intramuscular fat of raw and thermally treated beef, were C 18:1 *cis*, C 16:0, and C 18:0. They stated that, the thermal treatment affected on the fatty acid composition of beef. As a result of steam cooking, the relative proportion of C 18:2 *n-6* and C 18:3 *n-3*, increased in comparison to the raw beef, whereas steam cooking and S-V treatment increased the proportion of C 20:4 *n-6*. Steam cooking decreased the proportion of Ʃ SFA, and increased the proportion of Ʃ PUFA *n-6*, and Ʃ PUFA *n-3* compared to the raw and S-V samples. There were no significant differences in percentage of Ʃ SFA and Ʃ MUFA in S-V and raw beef muscles. In our experience the percentage of Ʃ SFA for M, S-V and S goose meat was higher (no significant differences were for S-V and M meat and S-V>S) than in raw meat, but there were no significant differences in percentage of Ʃ MUFA compared to raw sample. The Ʃ PUFA percentage including Ʃ PUFA *n-6*, Ʃ PUFA *n-3* and Ʃ PUFA *n-6*/*n-3* ratio were in steamed and S-V cooked beef higher than in raw meat, but there were no significant differences in case S-V technique. Sous-vide beef had a lower proportion of Ʃ PUFA including Ʃ PUFA *n-3* than steam-cooked meat. In S-V goose meat, the proportion of Ʃ PUFA *n-6* and Ʃ PUFA *n-3* were lower, but Ʃ PUFA *n-6*/*n-3* ratio was higher than in raw meat and differed in comparison mainly to M meat. The AI and TI lipid indices (calculated on the basis of data provided by Modzelewska-Kapituła et al. (2019) for S-V (0.88; 1.72 respectively) and steamed (0.74; 1.59 respectively) beef were similar to raw meat (0.82; 1.77 respectively). The AI index was lower, but TI was much higher than the recommended. In our study the AI and TI slight increase for S-V, M, S compared to raw meat and the AI were lower but TI slightly higher than the adequate. Also, the calculated NVI and Ʃ DFA/Ʃ OFA ratios for both heating techniques in raw beef meat were similar. Likewise, the NVI and Ʃ DFA/ƩOFA ratios for S-V, M, S and raw goose meat were similar. Similarly, to our study, Rasinska et al. (2019) observed increase in Ʃ SFA and decrease in Ʃ PUFA proportion in S-V (72.5°C for 2.5 h) rabbit meat compare to raw ones. These differences were lower than in roasted (R) meat. They related that the C 16:0 was lower by 7.16% in S-V meat, and in contrast the C 18:0, C 14:0 were significantly higher (by 13.3; 59.7% respectively) than in raw sample. In our experiment the percentage of C 18:0 in S-V goose meat were higher than raw meat (by 38.2%) but C 14:0 similar. The Ʃ MUFA decreased slightly in boiled and roasted rabbit meat but in S-V and raw meat were similar. Boiling, roasting and S-V cooking decreased the content of C 18:1 in rabbit meat, while the C 16:1 increased in S-V meat but decreased in boiled and roasted compared with raw meat. In our study Ʃ MUFA in S-V meat increased (mainly C 18:1 *cis*) compared to raw meat. Sous-vide method resulted in a lower loss of C18:2 (by 3.3%) in rabbit meat, than roasting (by 9.5%). An even more pronounced effect of roasting they found for C18:3 (by 46.3%) whereas S-V cooking caused lower decrease of C 18:3 (by 12.6%). The consequence of the increase in Ʃ SFA and the decrease in Ʃ PUFA in S-V rabbit meat were higher values of indices such as: Ʃ PUFA *n-6*/*n-3*, AI, TI (7.79; 0.79; 0.98 respectively), compared to raw meat (7.0; 0.66; 0.90 respectively). The trends of our results were in line with those. The TI and Ʃ PUFA *n-6*/*n-3* indices were higher than the recommended ones, while the AI was similar. Thermal treatment (all methods) did not change the amount of TFA and the Ʃ DFA/Ʃ OFA ratios, while it caused a decrease in the values of the following indices: HPI, UI, PI, IB, Ʃ UFA/Ʃ SFA, Ʃ PUFA/Ʃ SFA. The decrease in IB can promote positive health effects. Also, Głuchowski et al. (2020) stated that the FAs profile in chicken breast's depended on the type of heat treatment and its parameters (time and temperature). They show, that S-V cooking (S-V64 = 64°C, 60 min; S-V66 = 66°C, 80 min; S-V75 = 75°C, 35 min) no changed the share of Ʃ SFA compared to raw meat, but the boiled and steamed meat was characterized by a higher proportion of Ʃ SFA than raw material and S-V samples. It resulted mainly from a statistically significant (*P* ≤ 0.05) higher percentage of C 14:0 and C 18:0. The group of SFA was dominated by C 16:0, whose share slightly increased in all (except S-V64) methods of heat treatment, too. The share of Ʃ MUFA were a statistically significant higher in the S-V samples for the longest cooked (S-V66). The proportion of C 18:1 *cis* was statistically significantly the highest in the S-V66 samples (36.4 v/s 31.6-33.5 g/100 g FAs). Only S-V64 meat was characterized by a similar share of Ʃ PUFA to raw meat, while other types of heat treatment caused its significant losses. Among Ʃ PUFA, the highest identified was C 18:2 *n-6*, which in condition S-V64 did not change compared to raw meat, but the remaining types of heat treatment caused its significant decrease. Głuchowski et al. (2020) that, the S-V chicken breast using the lowest S-V64 temperature had the most similar FAs profile to the raw material. This sample particularly was characterized by a higher content of Ʃ PUFA *n-6* and Ʃ PUFA *n-3* and the ratio of these acids (9.83) was close to the raw material (9.42), while other were higher (10.24-11.76). All data of Ʃ PUFA *n-6*/*n-3* ratio were no consistent with suggested as suitable. Also, indicators such as: Ʃ UFA/Ʃ SFA (1.96), Ʃ PUFA/Ʃ SFA (0.79), Ʃ DFA/Ʃ OFA (3.09), HPI (1.85), UI (96,19%) PI (34.32%) were significantly higher for SV64 meat, while AI (0.54), TI (0.74) lower than for the other samples, but close to the value for raw meat. The higher value of Σ UFA/Σ SFA, Σ DFA/Σ OFA, HPI for S-V64 compared to remaining samples is more beneficial for human health. While higher values of UI and PI indicators testify about higher susceptibility to auto-oxidation of FA in S-V64 meat, but higher protective potential for CVD compared to the remaining ones, too. The Σ PUFA/Σ SFA ratio, and AI were consistent with advised as good values and TI slightly higher. The nutritional value of the NVI was also slightly lower (1.98) than this in raw meat (2.04) (all lipid indexes are calculated on data given by Głuchowski et al. (2020). Our results for S-V meat are partially in line with those for longer time of heat treatment (66°C, 80 min.). The effect of S-V technique on fatty acid composition in beef meat was the subject of research by (Falowo et al., 2017). There were no significant differences (*P* > 0.05) in share of Σ SFA, Σ MUFA and Σ PUFA by application of S-V cooking method at 65°C (120min.) and 85°C (60min.) (S-V65 and S-V85 respectively). However, the proportion of Ʃ SFA, Ʃ MUFA, lipid indices such as AI, and Σ PUFA *n-6*/*n-3* were lower in S-V85 and raw meat than in S-V65 samples. Also, percentage of Σ PUFA, Σ PUFA *n-6*, Σ PUFA *n-3* and lipid indices Σ PUFA/Σ SFA, Σ PUFA/Σ MUFA, were higher at 85°C and in raw meat than at 65°C cooking temperature.

The results of Echarte et al. (2003) indicate that microwave heating significantly changed the FAs profile of chicken patties compared to raw products. Similar to our results for microwave cooking, they stated an increase in Ʃ SFA which was primarily the result of changes in C 16:0 and C 18:0. Unlike our results, the proportion of Ʃ MUFA in chicken patties (mainly C 16:1) increased but of Ʃ PUFA did not change compared to raw products. The use of microwave heating did not modify the indices such as:Ʃ PUFA *n-6/n-3*, NVI, Ʃ UFA/Ʃ SFA, AI, HPI, PI, CB in chicken meat. In microwaved chicken meat decreased in Ʃ PUFA/Ʃ SFA, Ʃ DFA/Ʃ OFA, UI, while increased in TI and IB indexes compare to raw material. Microwave cooking particularly modified the IB and this was about a 3-fold increase compared to fresh meat. This meat was very unfavorable for humans’ health. In our study microwave cooking did not modify only Ʃ DFA/Ʃ OFA and NVI indices, and remaining changed showing upward (Ʃ PUFA *n-6/*n*-3*, AI, TI, IB) or downward (Ʃ UFA/Ʃ SFA, Ʃ PUFA/Ʃ SFA, PI, CB, UI, HPI) trends. Maranesi et al. (2005) reported that microwave cooking modified the concentrations of some FA in lamb rib-lions significantly compared to the uncooked samples. Some SFA increased significantly, namely C 14:0, C 15:0, C 16:0, but the ƩSFA increased, without any statistical differences. The Ʃ MUFA and Ʃ PUFA (C 20:4 and C 18:2) decreased slightly, but there were no significant differences compared to raw lamb loins. They noted a significant increase of C 18:1 *trans* concentration compared to the raw samples. In turn, comparing microwave cooking and broiling, in both cooking techniques the Ʃ PUFA *n-6/n-3* (10.9, 11.0 respectively) did not change and the Ʃ PUFA/Ʃ SFA (0.18, 0.18 respectively) decreased, but no significantly compared to raw meat. There were no significant differences between microwave cooking and broiling in the Ʃ DFA/Ʃ OFA (2.15, 2.17 respectively), Ʃ UFA/Ʃ SFA (1.09, 1.09 respectively), NVI (1.90, 1.92 respectively), AI (0.82, 0.81 respectively), TI (1.62, 1.63 respectively), PI (15.94, 15.87 respectively), and HPI (1.21, 1.22 respectively) values. Whereas, the raw meat compared with microwaved, had higher Ʃ UFA/Ʃ SFA (1.13), Ʃ DFA/Ʃ OFA, (2.27), PI (16.78) ratios, lower value of AI (0.77) and TI (1.60) indexes and higher health promoting index HPI (1.29) (calculated on the basis of data given for lamb meat by Maranesi et al. 2005). Both methods, microwave cooking and broiling worsened these lipid indicators. In their study, the indexes: Ʃ PUFA *n-6/n-3*, TI were higher than recommended and Ʃ PUFA/Ʃ SFA lower. The results obtained in our experiments for goose meat subjected microwave cooking are partly, similar to the previous data presented by Alfaia et al. (2010) for beef meat. The authors reported an increase of Ʃ SFA (mainly C 14:0, C 16:0, C 17:0 and C 18:0) in comparison to the uncooked meat control. They observed a significant increase in the relative proportion of Ʃ MUFA (by 1.9%) which was the consequence of increase in C18:1 *cis*. On the other hand, cooked beef had lower concentrations of Ʃ PUFA (by 5.8%) than raw meat, due to a significant loss of some *n-6* and *n-3* PUFA. The microwave cooking generated the formation of TFA, but compared to raw meat differences were not significant. The cooked samples were characterized by the lower value of the Ʃ UFA/Ʃ SFA, Ʃ PUFA/Ʃ SFA, Ʃ DFA/Ʃ OFA, HPI, UI, PI, CB and higher NVI, IB indices compared to raw meat. The Ʃ PUFA *n-6/n-3* relation was similar to those for raw samples. In our work, the Ʃ PUFA *n-6/n-3* ratios for M, S-V and S samples were higher than for raw meat. In their study, for microwaved beef meat, the Ʃ PUFA/Ʃ SFA was low (0.44), but together with the AI index (0.56) were close to the recommended limit, while TI (1.12) (calculated based on the provided data) was higher. The microwave cooking did not worsen the nutritive index (NVI), and our data concerning M, S-V and S goose samples were in line with those. In our study the all-cooked samples had a lower protective potential (PI) for coronary artery disease, higher inflammatory biomarker indexes (IB), lower CB than raw ones. These results are in agreement with the data obtained by Alfaia et al. (2010), for beef meat. Our results for microwaved Polish goose meat were partly in line with those found by Oz and Celik (2015) for Turkish goose meat. In our study, the Ʃ SFA in microwaved breast muscle (31.12%, *P*=0.001) was similar to value stated for Turkish microwaved goose meat (32.06%). Like as in our study the Ʃ SFA percentage in cooked meat was higher than in raw ones. The increase in the relative proportion of Ʃ SFA, which occurred after cooking, mainly resulted from an increase in C 18:0. But opposite to our results the percentage of C 16:0 was lower in microwaved meat compared to raw sample. They observed a slight decrease in the percentage of Ʃ MUFA, but there was no significant difference with data for raw meat. In our research, we have not indicated significant differences in the sum of MUFA for raw and microwaved samples, too. On the contrary to results Oz and Celik (2015) we established the lower value for Ʃ PUFA including Ʃ PUFA *n-6* and Ʃ PUFA *n-3* for cooked meat. In Turkish cooked goose meat the Ʃ PUFA/Ʃ SFA increased and Ʃ PUFA *n-6/n-3* decreased, wherein both indicators corresponded to the recommended values. In turn, in our experiment, this was reversed, and Ʃ PUFA *n-6/n-3* was much higher than adequate value. Similar to our results Ʃ UFA/Ʃ SFA ratio was slight lower in microwaved Turkish goose meat than raw ones. The Ʃ DFA/Ʃ OFA, AI, UI, PI, NVI (calculated on the basis of the given values), indicators achieved similar values to Polish goose meat. On the other hand, the TI (0.59) and IB (0.66) indices for cooked meat were lower than our results (0.65; 2.97, respectively, *P* = 0.001), which was more favorable from the dietary point of view. Campo et al. (2013) observed that the fatty acid profile of meat affected by the cooking method, too. In contrary to our results for S goose meat the Ʃ SFA for stewed (S) lamb meat decreased compared to raw, roasted (R), grilled (G) samples (by 16.4% 17.1%, 14.1% respectively). While Ʃ MUFA and Ʃ PUFA increased as in our experiment (there were no significant differences). Among major FA in lamb meat, the largest differences between treatments appeared for C 16:0 and C 18:2 *n-6.* The percentage of C 16:0 largely decreased in S vs. G, R or the raw meat with an 15.3% decrease in relation to the raw sample. Although not significant C 18:0 also showed a decreased of 15.1% in comparison with the raw product. The percentage of Ʃ PUFA followed the opposite tendency, in higher proportion in S than in raw, R or G meat, mainly due to the share of Ʃ PUFA *n-6,* in which C 18:2 *n-6* was the major. No significant differences were found in any individual PUFA *n-3* or in the group of all Ʃ PUFA *n-3* FA. As a consequence, S meat showed a very high Ʃ PUFA *n-6*/*n-3* index (20.88 v/s 8.64−9.47), far away from the recommendations, higher unsaturation index (UI) (72.3 v/s 58.9−60.43%) index which testifies a higher susceptibility to oxidation during technological processes. The S lamb had a more favorable Ʃ UFA/Ʃ SFA (1.47v/s 1.03−1.10), Ʃ PUFA/Ʃ SFA ratios (0.35) than the rest of samples (0.17), which was lower than desirable 0.45. The Ʃ DFA/Ʃ OFA (2.84), HPI (1.62) indexes were higher in the S meat than in the remaining ones (2.05−2.16; 1.04−1.13 respectively). The better values of AI (0.43 v/s 0.60−0.65) and TI (4.71 v/s 7.33−7.94) was also found in the S lamb, it showed a decreased of 31% and 41% (respectively) in relation to the raw meat and the other samples, but the TI ratio was far away from the suggested as suitable. The high value of the IB (12.33 v/s 8.55−10.75) and low values of PI index (18.19 v/s 13.24−13.63%) is also disturbing, which means that this meat should be included in the human diet occasionally. In our experiment the AI, TI, IB, UI, Ʃ PUFA/Ʃ SFA, Ʃ DFA/Ʃ OFA, HPI, PI indexes for S goose meat were more favorable from the health point of view than that for S lamb. In the study of Li et al. (2016) contrary to our results, the stewing (95°C, 150 min) resulted in a reduction in total SFA compared with raw pork belly. The decrease in C 16:0 and C 18:0 was the main contributor to the reduction in total SFA. It was shown that C 18:1 *n-9* and C 18:2 *n-6* were the main MUFA and PUFA in pork belly, while PUFA *n-3* could not be detected. The induction stewing treatment of pork meat did not result in greater total MUFA compared to raw sample. In our study all type of heat treatment (M, S-V, S) caused a slight increase (by 2.1−4.6%) in the proportion of C 18:1 *cis* in relation to raw material (*P* = 0.001). Li et al. (2016) stated that the stewing increased the Ʃ PUFA percentage by 13.3% compare with raw meat. In our experiment with goose meat, the Ʃ PUFA was slightly higher in S samples than in raw meat. The Ʃ PUFA/Ʃ SFA ratio (0.35) in S belly pork was lower than the recommended but higher than in raw meat (0.30). The induction stewing of pork meat caused lower AI, TI indexes (0.44, 5.79 respectively) compared to raw samples (0.48, 6.52 respectively), but the TI was much higher than advised as suitable. At the same time, taking into account the health benefits, after stewing, indicators such as: Ʃ UFA/Ʃ SFA, NVI, HPI, Ʃ DFA/Ʃ OFA, PI also improved in relation to raw meat.

It is difficult to discuss our results with the results of studies by other scientists, as they vary depending on the type of meat and the heat treatment conditions. There are no studies on goose meat in the literature, while those concerning other types of meat and different heat treatment conditions.

## CONCLUSION

Sous- vide, microwave cooking and stewing for both kinds of meat caused a significant increase in the Σ SFA proportion, Σ PUFA *n-6/n-3,* Σ PUFA/ΣSFA, AI, TI, IB ratios compared to raw meat. The lowest ratio of Σ PUFA *n-6/n-3* was shown by the S-V muscles, but all calculated values for Σ PUFA *n-6/n-3* were far from the recommended, and it was a phenomenon very unfavorable from a dietary point of view. While taking into account the Σ PUFA/Σ SFA, AI, TI indices, they were consistent with suggested as suitable or very close to expected value, good for human health. The cooking of meat with skin by S-V technique was more beneficial for consumers than the remaining methods in terms of Σ DFA/Σ OFA, Σ UFA/Σ SFA, NVI, AI, HPI, IB indexes, and Σ SFA, Σ OFA, Σ UFA, Σ DFA values (but not all of these were significant). In turn, stewing the meat without skin was more favorable than S-V and microwave cooking in terms of Σ DFA/Σ OFA, Σ UFA/Σ SFA, Σ PUFA/Σ SFA, PI, UI, AI, TI, HPI indexes.
